# HIV related risk behaviours among taxi drivers and their assistants in Addis Ababa, Ethiopia: descriptive cross-sectional survey

**DOI:** 10.1186/1471-2458-14-330

**Published:** 2014-04-08

**Authors:** Yihunie Lakew, Habtamu Tamene

**Affiliations:** 1 Ethiopian Public Health Association, Addis Ababa, Ethiopia; 2 Population Service International, Addis Ababa, Ethiopia

**Keywords:** HIV, Risky sexual behaviours, Taxi drivers and assistants

## Abstract

**Background:**

Risk taking behaviours in relation to HIV among the mobile population is a growing public health concern in many developing countries, including Ethiopia. The aim of this study was to describe risky sexual behaviours and associated factors among male taxi drivers and assistants in Addis Ababa.

**Methods:**

A descriptive cross-sectional survey design with multistage cluster sampling procedure was employed to select 615 individuals for interview.

**Results:**

Seventy six percent of the respondents were sexually active. Nearly 31% of the respondents reported casual sex and 7% of them did not use a condom with their most recent casual sex partner. More than half (58.5%) of the respondents had no condom use efficacy. Condom breakage and/or slippage during sex had been encountered by 44% of respondents with casual partners and sex during menstruation had ever occurred among 17% of respondents. Eleven percent had experienced sex with female sex workers. Thirty-three percent of the respondents were unfaithful to their spouse/steady partners. Multivariate analysis revealed that living with parents [AOR 95% CI; 2(1.14-3.60)], non-khat chewers [AOR 95% CI; 3.7(2.13-6.31)], never taken VCT [AOR 95% CI; 3.5(1.84-6.72)], middle-class monthly cash gain [AOR 95% CI; 0.5(0.25-0.98)] and more years of experience working on a taxi [AOR 95% CI; 0.17(0.60-0.47)] were statistically significant to influence lifetime abstinence. Non-khat chewers [AOR 95% CI; 0.53(0.37-0.78)], never taken VCT [AOR 95% CI; 0.54(0.36-0.88)] and higher monthly cash gain [AOR 95% CI; 2.9(1.14-7.19)] had a statistically significant association with condom use efficacy. Living with parents [AOR 95% CI; 2(1.31-3.72)], living with friends [AOR 95% CI; 6.4(3.13-12.89)] and non-khat chewers [AOR 95% CI; 2(1.34-3.53)] were risk factors found to be associated with faithfulness.

**Conclusions:**

Risky sexual behaviours in this sub-population were considerable and associated factors were found to be multidimensional. Therefore, there is a need for robust intervention strategies such as tailored serial radio program targeting taxi drivers and their assistants.

## Background

HIV/AIDS emerged in the 1980s as one of the major epidemics in the history of mankind [1]. Sub-Sahara African (SSA) countries remain most heavily affected by the epidemic. In 2011, an estimated 23.5 million [22.1–24.8 million] people were living with HIV in the region, which accounted for 69% of the global AIDS burden [2]. Ethiopia is one of the SSA countries most affected by the epidemic. Although reports indicate that the HIV epidemic is leveling off in Ethiopia, a significant number of people live with the virus and many families are affected [3,4]. With a population of nearly 74 million in 2007, and about 1.4 million cases, Ethiopia carries a heavy burden of disease. It is estimated that HIV is responsible for about a third of all adult deaths in the age group 15–49 years and leaves nearly three-quarters of a million orphans in the age group 0–17 years and over 30,000 newborns with the virus per year [5].

The HIV epidemic has penetrated almost all population groups, including the hard-to-reach rural areas. However, with the existing socio-cultural diversity of Ethiopia, the pattern and distribution of HIV in the country widely varies. Some regions are more affected than others. The epidemic is more prevalent in urban than rural communities [3,4]. Currently, the epidemic tends to concentrate in certain population groups who have risky sexual behaviours. Female sex workers (FSWs), long distance drivers, and daily laborers have been identified with high sexual risk behaviors for the epidemics of HIV infection in Ethiopia [6,7]. This suggests that there are key populations at risk of HIV exposure that may play a key role in the epidemics.

Research in the 1990s indicated that about 40% of taxi drivers were found to be at risk of HIV in Gondar, Northwest Ethiopia [6]. However, mini-bus-taxi drivers were not specifically counted in Ethiopia’s sentinel surveillance system [7,8]. Recent data about HIV risk behaviours and risk factors among taxi drivers is limited. A study done in 2005 by Steve Tarevella revealed that taxi drivers are vulnerable to HIV infection because they travel throughout the city during their work, encounter different people, receive money, and sometimes find themselves pursued by women who hope to have money spent on them by the drivers [9]. Use of khat, alcohol, and other stimulants are also common practices that motivate drivers to have unsafe sex [9].

In this regard, understanding risky sexual behaviours and the factors that drive or influence the epidemic among taxi drivers and their assistants is an important issue. Therefore, this study is aimed to describe risky sexual behaviorus and associated factors that result in HIV infection among male taxi drivers and their assistants in Addis Ababa.

## Methods

### Study design

A descriptive cross-sectional survey was conducted during the period of April 8–17, 2006.

### Sample size

For the determination of sample size the following attributes were considered: (1) The anticipated proportion of transport workers (inter-city bus drivers) that did not use a condom (~24%) during their most recent sexual encounters with casual partners [7]; (2) 95% confidence level (3) 5% worst acceptable result (precision) and (4) design effect of two. The sample size, including a 12% adjustment for the non-response was estimated at about 660 individuals.

### Sampling procedure

A multistage cluster sampling technique was employed to select the required study subjects. In the first stage, all available clusters (taxi areas or stations) within Addis Ababa limits were identified based on the usual taxi density. Each cluster contains more than two taxi stations or areas. A total of 12 major taxi stations/areas were selected as a sampling unit. In the second stage, eligible study subjects were randomly interviewed from 12 selected clusters (taxi stations/areas). Taxi drivers and their assistants at the age of 15 and above and who worked at least six months duration on taxi services were eligible for an interview.

### Data collection instrument

The data were collected using a structured questionnaire. The questionnaire was prepared in English and then translated to Amharic, which is a local language. The Amharic version of the questionnaire was pretested and the necessary modifications were made before the actual survey. Data collectors were second and third year students at Addis Ababa University with prior experience in data collection. Training was given to the data collectors on how to conduct the interview. Interviews were often completed during off-peak times when the taxi drivers and their assistants were less busy (i.e. mostly after 9:00 am in the morning and before 5:00 pm in the afternoon).

### Data management and analysis

The data were entered into a computer system using SPSS version 15.0 and analyzed using STATA version 11. Appropriate statistical methods ranging from simple descriptive statistics to multivariate logistic regression analysis were employed to describe risky sexual behaviours and associated socio-demographic and psychosocial factors. Bivariate analysis was used to identify the relative importance of each predictor to the dependent variables by controlling the effects of other variables. Those variables which were significant on bi-variate analysis at (P-value < 0.05) were entered to multivariable logistic regression analysis to examine the effect of each independent variable to the dependent variables of the study [10].

### Variables

If respondents engaged in any form of sexual activities, it was coded as 1, otherwise lifetime abstinence was coded as 0. Faithfulness, defined as respondents that never had sex with anyone other than their wives or steady partners was coded as 1 and otherwise 0. Condom use efficacy was a binary composite score outcome response that derived ability from questions of condom use during sex with casual sex partners, and were the dependent variables. Potential socio-economic, demographic and psychosocial factors including education (cannot read and write, grades 1–4, 5–8, 9–12, 12+), type of taxi drivers (mini-bus-taxi drivers, assistants, Lada taxi drivers), alcohol use (no, yes), age (15–19, 20–24, 25+), khat chewing habit (no, yes), usual taxi working areas (central areas of the city, peripheral areas), taxi ownership status (own taxi, employed), living arrangement (alone, with parents/relatives, with girlfriend/peers), monthly cash gain from taxi driving in birr (210–435,436-630,631-880,881-3640), and years of experience driving a taxi (<1 year, 1-5 years, 5+ years) were considered as independent variables. Most of the dependent and independent variables were adopted from previous behavioural surveillance surveys [6,7] conducted in Ethiopia and research conducted elsewhere [8,11]. Lada taxi drivers are those who give transportation services both during day and night times with 4 people at a time whereas mini-bust taxi drivers are mostly working during day time with 12 people at a time.

### Ethical issues

Ethical clearance was obtained from Addis Ababa University, Institute of Development Research and Population Studies Research Centre. Participation in the interview was entirely based on informed consent. No personal identifiers of the respondents were recorded.

## Results

### Socio-demographic characteristics

A total of 615 individuals were interviewed, yielding a 93.2% response rate. More than half of the study participants were both mini-bus-taxi drivers and Lada taxi drivers. The mean age and mean sexual debut of the study population was 26 (SD 6.06) and 18.3 (SD 2.4) years, respectively. The majority (90%) of the respondents were under the age of 35 years old with a range of 15–45 years. Educational status was nearly universal in this sub-population with 96.1% having ever attended formal education. Only 17.2% of the respondents were currently married at the time of the survey. Orthodox Christianity was the predominant religion (72.4%) reported, followed by Muslim (20.7%) and last, other denominations of Christianity (6.7%). More than half (62.6%) of the respondents have lived in Addis Ababa since birth, with about 46% currently residing with their parents. The largest proportions (80.3%) of the respondents were employed taxi drivers, while the remaining (19.7%) either owned their taxi or worked on taxis owned by their families. Fifty-six percent of the respondents have worked on the taxis for 1–5 years. Taxi drivers’ monthly cash gain varies in accordance with their taxi ownership status (Table 1).

**Table 1:** Background characteristics of taxi drivers and assistants in Addis Ababa, 2006

**Background variables**	**Freq**	**Percent**
**Categories of taxi drivers**		
Minibus driver	201	32.7
Assistants	251	40.8
Small taxi (‘Lada’) drivers	163	26.5
**Age**		
15-19	76	12.4
20-24	195	31.7
25-34	280	45.5
35+	64	10.4
**Education**		
Can’t read and write	24	3.9
Grade 1-8	260	42.3
Grade 9-12	289	47
Grade 12+	42	6.8
**Marital status**		
Single	465	75.6
Ever married	150	24.4
**Religion**		
Orthodox	445	72.4
Muslim	127	20.7
Others	41	6.7
**Place of birth**		
In Addis Ababa	381	62
Out of Addis Ababa	234	38
**Experience on taxi**		
<1 year	45	7.3
1-5 years	346	56.3
5 + years	224	36.4
**Taxi ownership**		
Yes	121	19.7
No	494	80.3

### Risk behaviours among the respondents

Table 2 shows that almost all of the study subjects had previously received information on HIV/AIDS. The majority of the respondents (76.4%) were sexually active preceding the survey. Only 23.6% of the participants have abstained in lifetime from any form of sex at the time of the survey. About 30.6% of the respondents had casual sex in 12 months prior to completion of the survey. Of those who had casual sex, 6.9% of the respondents did not use a condom with their casual sex partners during their most recent intercourse. Nearly 11% of the respondents have had sex with female sex workers (FSWs). All taxi drivers and their assistants who had sex with a FSW reported that they utilized condom. Of those who ever used condoms, around 44% encountered condom breakage and/or slippage and 16.8% reported that they have had sex during menstruation with their casual sex partners at some point in their lifetime. About 33% of the respondents were unfaithful to their spouse/steady partners. The number of lifetime sexual partners in all respondents ranged from 1–80. In the 12 months preceding the survey, the average number of partners ranged from 1–8. The mean number of lifetime sexual partners and 12 months prior to the survey was 7.1 (SD 10.6) and 1.6 (SD 1.14), respectively. The median age at first sex was at about 18 years (SD 2.4).

**Table 2:** Level of risky sexual behaviours among taxi drivers and assistants in Addis Ababa, 2006

**Variables**	**(n = 615)**	**Percent (95% CI)**
Abstinence in lifetime		
Yes	145	23.6 (20.21-26.94)
No	470	76.4 (73.10-79.79)
Faithfulness with spouse/steady partner		
Yes	289	67.5 (63.07-71.98)
No	139	32.5 (28.02-36.93)
Casual sex in the last one year		
Yes	131	30.6 (26.22-34.99)
No	297	69.4 (65.01-73.78)
Condom use with casual sex at last sex		
Yes	122	93.1 (88.74-97.52)
No	9	6.9 (2.48-11.26)
Ever had sex in lifetime with casual partners while menstruating		
Yes	22	16.8 (10.31-23.28)
No	109	83.2 (76.72-89.69)
Ever encountered condom breakage or slippage in lifetime with casual partners		
Yes	58	44.3 (35.66-52.89)
No	73	55.7 (47.11-64.34)
Had sex with FSWs in the last one year		
Yes	48	11.2 (8.21-14.23)
No	380	88.8 (85.78-91.79)

### Factors associated with HIV related risky sexual behaviours

As shown in Table 3, the logistic regression model revealed that monthly cash gain had an inverse relation with lifetime abstinence. Drivers and assistants who gained monthly cash flow of 436–630 birr (second quintile) were 50% less likely to have lifetime abstinence compared with the first quintile (210–435 birr). VCT uptake and khat chewing habit had an association with lifetime abstinence. Taxi drivers who had no experience in VCT uptake were about five times more likely to have the experience of lifetime abstinence than those who ever had experience of VCT uptake. On the other hand, drivers who never chewed khat were nearly four times more likely to have the experience of lifetime abstinence than those who ever had khat chewing habit. Living arrangement also had association with experience of lifetime abstinence. Taxi drivers who lived with their parents/relatives were about twice more likely to have experience of lifetime abstinence than those who lived alone. Assistants of taxi drivers were four times more likely to have experience of lifetime abstinence than minibus taxi drivers. Experienced workers on taxi were less likely to abstain than fresh workers. Other variables including alcohol, taxi ownership status and long year experience on taxi appeared to have association with lifetime abstinence in the bivariate step, but disappeared at logistic model. Age group was adjusted with lifetime abstinence, controlling its effect at this level of analysis.

**Table 3:** Factors associated with experienced in lifetime abstinence as HIV risk preventive behaviour in taxi drivers and assistants, 2006

**Variables**	**Crude OR 95% CI**	**Adj OR 95% CI**
**Categories of taxi drivers**		
Minibus taxi	1	1
Assistant	5.4 (3.26-8.90)	4 (1.58-9.45)*
Small taxi	1 (0.54-1.95)	0.76 (0.35-1.65)
**Khat**		
Yes	1	1
No	3.9 (2.48-6.04)	3.7 (2.13-6.31)**
**Living arrangement**		
Alone	1	1
With parents/relatives	2.4 (1.48-3.78)	2 (1.14-3.60)*
With girlfriend/peers	0.47 (0.23-0.99)	0.60 (0.25-1.42)
**VCT**		
Yes	1	1
No	4.7 (2.75-8.08)	3.5 (1.84-6.72)*
**Monthly cash gain**		
210-435	1	1
436-630	0.30 (0.18-0.50)	0.50 (0.25-0.98)*
631-880	0.19 (0.12-0.31)	0.83 (0.34-2.04)
881-3640	0.08 (0.036-0.19)	0.31 (0.08-1.25)
**Experience on taxi**		
<1 year	1	1
1-5 years	0.86 (0.45-1.64)	1.1 (0.50-2.39)
5 + years	0.07 (0.0281-0.17)	0.17 (0.60-0.47)*

*P < 0.05 ** P < 0.001.

As shown in Table 4, higher income from taxi work had positive association with condom use efficacy. Taxi drivers who had a better monthly cash gain were more likely to be able to use condom during casual sexual practices. On the other hand, non-VCT users and non-khat-chewers have had inverse association with condom use efficacy. Taxi drivers who had never used VCT services were less likely to have the experience of condom use efficacy than those who had used VCT services. Taxi drivers who never chewed khat were nearly 50% less likely to have condom use efficacy than those who ever chewed khat. Educational status and type of taxi drivers were found to be significant at bivariate analysis but not in multivariate analysis.

**Table 4:** Factors associated with condom use efficacy as HIV risk preventive behavior in taxi drivers and assistants, 2006

**Variables**	**Crude OR 95% CI**	**Adj OR 95% CI**
**Khat**		
Yes	1	1
No	0.63 (0.45-0.87)	0.53 (0.37-0.78)*
**VCT**		
Yes	1	1
No	0.53 (0.37-0.76)	0.54 (0.36-0.80)*
**Monthly cash gain**		
210-435	1	1
436-630	1.8 (1.14-2.82)	1.7 (0.96-2.94)
631-880	3.2 (2.06-4.85)	3.1 (1.49-6.44)*
881-3640	2.6 (1.56-4.28)	2.9 (1.14-7.19)*

*P < 0.05 ** P < 0.001.

As shown in Table 5, non-khat-chewing habit and living with parents friends were found to have positive association with faithfulness. Participants who never had a khat chewing habit were 2 times more likely to be faithful to their steady sexual spouses than khat chewers. Taxi drivers and assistants who lived with their parents or relatives and those who lived with girlfriends/peers were about twice and 6.4 times more likely to be faithful to their steady sexual spouses than those who lived alone, respectively. Participants who had never used VCT service were about 65% less likely to be unfaithful for their steady sexual partners than those who ever taken VCT. Alcohol appeared to have a statistically significant association with faithfulness in the bivariate step but was not in the logistic analysis.

**Table 5:** Factors associated with faithfulness as HIV risk preventive behaviour in taxi drivers and assistants, 2006

**Variables**	**Crude OR 95% CI**	**Adj OR 95% CI**
**Khat**		
Yes	1	1
No	2.2 (1.48-3.40)	2 (1.34-3.53)*
**Living arrangement**		
Alone	1	1
With parents/relatives	2.3 (1.47-3.73)	2 (1.31-3.72)*
With girlfriend/peers	6.6 (3.44-12.57)	6.4 (3.13-12.89)**
**VCT**		
Yes	1	1
No	0.33 (0.20-0.52)	0.35 (0.21-0.59)**

*P < 0.05 ** P < 0.001.

## Discussion

This study explored the risky sexual behaviours and associated factors among taxi drivers and their assistants. A number of risky sexual behaviours including casual sex, condom slippage, sex during menstruation, number of partners and age at first sex were identified in this sub-population group. This study’s findings were evidenced by a study conducted in four urban SSA that revealed the risk of HIV infection increased in casual sex and number of lifetime sexual partners [11]. A significant proportion of this study’s participants had sex with female sex workers (11.2%), which is higher than (7.9%) a study done among rickshaw pullers in Bangladesh [12]. This difference could be due to regional contexts as well as methodological and time variations between the present and previous studies. A higher proportion of the study participants had sex with casual partners while menstruating. This finding is consistent with a study done in the United States that associated with sexual intercourse during menstruation, regular and frequent sexual intercourse, and large number of sex partners as components of the transmission dynamics of STD including HIV [13]. Condom breakage or slippage in lifetime sexual practices of taxi drivers and assistants was also remarkably high. Compared with other target groups, casual sex practice in this study group was a two-fold increase from 15% in a cohort study among factory workers in Ethiopia [14]. However, with the same target population, the report of casual sex practices (33.3%) is similar to other developing countries [15]. Early sexual debut is another important individual-level risk factor identified in Zimbabwe for HIV infection among women [16]. Similarly, this study identified a large proportion of taxi drivers and assistants that did not delay the first sexual debut in their lifetime. The median age at first sex, in this study group, was similar with the study in Tanzania [17] that identified as age of first sex as one of the risk behaviours for HIV infection among youngsters. Furthermore, there was a wide variety of lifetime sexual partners reported from taxi drivers and assistants, which significantly increases their risk of contracting HIV. This finding was a two-fold increase from the results of lifetime sexual partners (1–40) reported in a former study conducted among 480 naval (military) personnel in Lagos, Nigeria [18] in which the difference could be explained by variations in time, target population and methodological approaches between the former and present studies.

Despite having such risky sexual behaviours, this sub-population was not included in the national behavioural surveillance study in Ethiopia [19] or by other researchers. Even if various governmental and non-governmental organizations have made efforts to achieve behaviuour change through mass-media exposure, community sensitization and outreach programs in promoting safe sexual practices to the general population, the adoption of preventive behaviours among male taxi drivers is relatively low. Many are still engaging in unprotected sexual activities. Institutions working for the prevention and control of HIV/AIDS had limited information about taxi drivers and their assistants and consequently failed to consider this high-risk group as an important sub-population for intervention in the country.

Socio-demographic factors independently associated with HIV risk behaviours were types of taxi drivers, living arrangements, monthly cash gain and taxi driving experience. This study recognized that monthly cash gain has a two-way association with sexual risky behaviours: 1) it has a negative relation with lifetime abstinence and 2) a positive association with condom use efficacy. Alongside with these socio-demographic factors, a number of psychosocial and behavioural related factors were also found to predict risky sexual behaviours. For this group of population, less exposure to VCT service had an association with unfaithfulness to their sexual partners and reduced the ability to use condom during sex with casual partners. This indicates that VCT service provision has been missed as an important strategy for HIV prevention for this population group in the country. Whereas, less exposure to VCT services had association with lifetime abstinence that possibly due to most people have been initiated to have VCT during the time of marriage.

Substance use is generally believed to be one of the associated factors for sexual risk behaviours in HIV transmission. Khat is a locally produced psycho-stimulant commonly used in Ethiopia. Khat is widely consumed among youth in the city. A study assessing the magnitude of sexually transmitted infection (STI) together with self-reports of sexual risk behaviour among youths (15–24 years old) in Addis Ababa, Ethiopia, reported that increased sexual activity was significantly associated with khat consumption [20]. Similarly, khat chewing habit among taxi drivers was also associated with unfaithfulness to sexual partners and early sexual initiations. However, khat chewers in this population group showed a higher condom use efficacy than non-chewers which might be due to prior knowledge and skills accumulated among the chewers.

The study population is extremely mobile which makes it difficult to properly classify clusters of taxi stations. Taxi working routes anywhere in the city and stations might have also potentially led to affect the homogeneity of clusters in this sup-population group. Furthermore, the study was a cross-sectional survey in its design which makes it impossible to determine causal relationships between the predictors and risk sexual behaviours. The self-reported measurements that were used might also have inherent biases and the potential for both underreporting and/or over-reporting in risky sexual behaviours. Despite these limitations, however, the study is important to give clue for further researches in similar population.

## Conclusions

This study documented higher risk sexual behaviours of taxi working populations. The findings showed that monthly cash gain, living arrangements, categories of taxi drivers, taxi driving experiences and khat chewing habits were likely to significantly influence the practice of adopting HIV/AIDS preventive behaviours. Targeted interventions tailored to taxi drivers as mobile population are recommended to reduce substance use habits and to protect their sexual risk behaviours.

## Competing interests

We declare that we have no competing interests in relation to this manuscript.

## Authors’ contributions

YL conceived the study, analyzed the data and drafted the report. HT contributed to the draft and review of the article. Both authors read and approved the final manuscript.

## Pre-publication history

The pre-publication history for this paper can be accessed here:

http://www.biomedcentral.com/1471-2458/14/330/prepub
